# C-type lectin Langerin is a β-glucan receptor on human Langerhans cells that recognizes opportunistic and pathogenic fungi

**DOI:** 10.1016/j.molimm.2009.12.016

**Published:** 2010-03

**Authors:** Marein A.W.P. de Jong, Lianne E.M. Vriend, Bart Theelen, Maureen E. Taylor, Donna Fluitsma, Teun Boekhout, Teunis B.H. Geijtenbeek

**Affiliations:** a Center for Experimental and Molecular Medicine, Academic Medical Center, University of Amsterdam, Meibergdreef 9, Amsterdam, The Netherlands; b Department of Molecular Cell Biology and Immunology, VU University Medical Center, Amsterdam, The Netherlands; c CBS Fungal Diversity Center, Utrecht, The Netherlands; d Division of Molecular Biosciences, Department of Life Sciences, Imperial College, London, UK

**Keywords:** β-Glucan, C-type lectin, Fungi, Langerhans cells, Langerin, Yeast

## Abstract

Langerhans cells (LCs) lining the stratified epithelia and mucosal tissues are the first antigen presenting cells to encounter invading pathogens, such as viruses, bacteria and fungi. Fungal infections form a health threat especially in immuno-compromised individuals. LCs express C-type lectin Langerin that has specificity for mannose, fucose and GlcNAc structures. Little is known about the role of human Langerin in fungal infections. Our data show that Langerin interacts with both mannan and β-glucan structures, common cell-wall carbohydrate structures of fungi. We have screened a large panel of fungi for recognition by human Langerin and, strikingly, we observed strong binding of Langerin to a variety of *Candida* and *Saccharomyces* species and *Malassezia furfur*, but very weak binding was observed to *Cryptococcus gattii* and *Cryptococcus neoformans*. Notably, Langerin is the primary fungal receptor on LCs, since the interaction of LCs with the different fungi was blocked by antibodies against Langerin. Langerin recognizes both mannose and β-glucans present on fungal cell walls and our data demonstrate that Langerin is the major fungal pathogen receptor on human LCs that recognizes pathogenic and commensal fungi. Together these data may provide more insight in the role of LCs in fungal infections.

## 1 Introduction

Langerin (CD207) is a C-type lectin expressed in human exclusively by Langerhans cells (LCs), which constitute a subset of dendritic cells that are located in epithelium of mucosal tissues and epidermis (Fithian et al., 1981; Patterson et al., 2002). LCs play a key role in the induction of immune responses against invading pathogens by capturing and processing foreign antigens and migrating to draining lymph nodes to present processed antigens to T cells (Banchereau and Steinman, 1998). As a C-type lectin, Langerin is thought to play a role in pathogen recognition by facilitating pathogen uptake and processing for antigen presentation (Hunger et al., 2004). Langerin is a type II transmembrane protein that contains one calcium-dependent carbohydrate recognition domain with a short cytoplasmic tail with a proline rich motif (Valladeau et al., 2003). Langerin forms a trimer on the cell surface and upon crosslinking with either a cell-bound or a soluble ligand, Langerin induces the formation of Birbeck granules, which are LC-specific intracellular organelles that appear as tennis-racket like structures with a size of <1 μm in diameter that are thought to be part of the endosomal recycling pathway (Valladeau et al., 2000).

Although Langerin is characterized as a pathogen recognition receptor, only few pathogens have been demonstrated to interact with Langerin. Both HIV-1 (de Witte et al., 2007; Turville et al., 2001) and *Mycobacteria leprae* (Hunger et al., 2004) have been identified as pathogens that interact with human Langerin, whereas murine Langerin was shown to bind to *Candida albicans* (Takahara et al., 2002, 2004). Langerin is important in protecting against HIV-1 transmission, since HIV-1 captured by Langerin is rapidly internalized into Birbeck granules for degradation, thereby preventing HIV-1 infection of LCs (de Witte et al., 2007; Turville et al., 2001). Based on the carbohydrate recognition specificity for mannose, fucose and N-acetyl-glucosamine monosaccharides (GlcNAc) (Stambach and Taylor, 2003), it is likely that Langerin has a broader specificity for pathogens than has been identified so far.

Fungal species are ubiquitous residents of human skin and many can cause invasive infections especially in immuno-compromised individuals. They can be acquired from the environment, such as *Cryptococcus neoformans*, or are part of the normal skin microbiota, such as *Candida albicans* (Richardson, 2005) or *Malassezia* species (Batra et al., 2005; Gupta et al., 2004). The close contact of the fungi with the epidermis and epithelial barrier suggests that LCs might be involved in fungal defense. Therefore we have investigated the interaction of a panel of fungi with Langerin on LCs.

Fungi, such as *Candida* species ubiquitously express β-glucans and mannosylated carbohydrate structures in their cell wall (Chaffin et al., 1998; Gantner et al., 2005). Here we demonstrate that Langerin not only recognizes mannose, fucose and GlcNAc structures, but also β-glucan structures. To our knowledge, thus far dectin-1 is the only C-type lectin in human that has been associated with β-glucan binding (Brown et al., 2003; Brown and Gordon, 2001). Here, we demonstrate that aside from dectin-1, Langerin also interacts with β-glucans, which can possibly mediate recognition of fungi and bacteria to initiate an anti-microbial response.

In addition, we demonstrate that Langerin is a receptor for *Candida* species, *Saccharomyces* species and *Malassezia furfur*, but only weakly interacts with *Cryptococcus gattii* and *Cryptococcus neoformans*. Our data further show that Langerin is the main C-type lectin receptor for fungi on primary human LCs even though these also express dectin-1. Thus, we have demonstrated that Langerin on LCs is a pathogen recognition receptor for β-glucans and a broad range of fungi. Increased knowledge of the pathogen recognition profile by Langerin on LCs can contribute to the understanding of the pathogenicity of fungi.

## 2 Results

### 2.1 Langerin recognizes *Candida*, *Malassezia* and *Saccharomyces* species

**Fig. 1:**
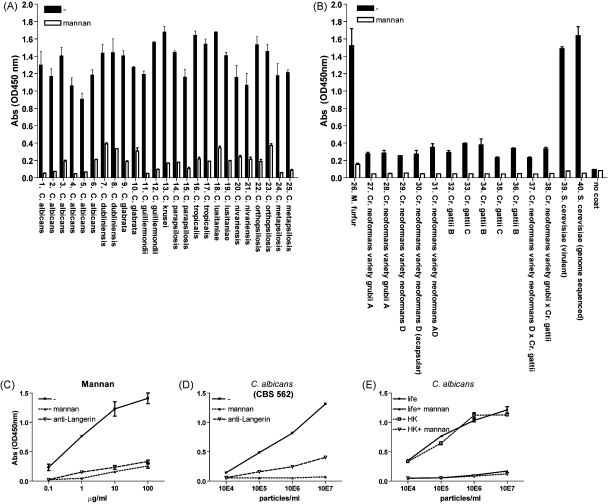
Langerin recognizes *Candida* species, *M*. *furfur* and *Saccharomyces* species. (A and B) Yeast strains were coated onto ELISA plates (10^7^/ml) and binding to recombinant Langerin was determined. Polysaccharide mannan was used to determine the specificity of the binding. (C and D) Polysaccharide mannan (C) and *C. albicans* (D) were coated onto ELISA plates at different concentrations. Both mannan and blocking antibody against Langerin (10E2) (de Witte et al., 2007) were used to determine specificity. (E) Binding of life and heat-treated *C. albicans* to Langerin. Data represent results from at least three independently performed ELISAs and was performed with two different batches of yeast strains. Error bars represent standard deviation of triplicates.

A large panel of pathogenic fungi was screened for binding to recombinant Langerin in an ELISA system (Table 1). This recombinant Langerin contains the whole extracellular region including the carbohydrate recognition domain and the coiled–coil stalk that allows oligomerization (Stambach and Taylor, 2003). To determine the specificity of the binding, polysaccharide mannan was used to inhibit the C-type lectin domain. Langerin interacted strongly with all *Saccharomyces* species, *Malassezia furfur* and *Candida* species, including *C. albicans*, *C. dubliensis*, *C. glabrata*, *C. guilliermondii*, *C. krusei*, *C. parapsilosis*, *C. tropicalis*, *C. lusitaniae*, *C. nivariensis*, *C. orthopsilosis* and *C. metapsilosis* (Fig. 1A and B). In contrast, no or low binding was observed for *Cr. neoformans* varieties and *Cr. gattii* of different serotypes (Fig. 1B). The inclusion of an acapsular *Cr. neoformans* (CBS 7935) demonstrated that the lack of binding is not due to the capsule.

To investigate the interaction between these fungi and Langerin in depth, a selection was made and a titration was performed. To determine the specificity of the Langerin binding ELISA, we investigated the ability of mannan and anti-Langerin to block the interaction of Langerin with coated ligands. Both inhibitors prevented Langerin binding to different concentrations of mannan and *C. albicans*, although mannan is slightly more efficient in blocking Langerin compared to anti-Langerin (Fig. 1C and D). In addition, both life and heat-killed *C. albicans* were compared and similar binding patterns were observed, demonstrating that heat-treatment did not alter the binding to Langerin (Fig. 1E).

**Fig. 2:**
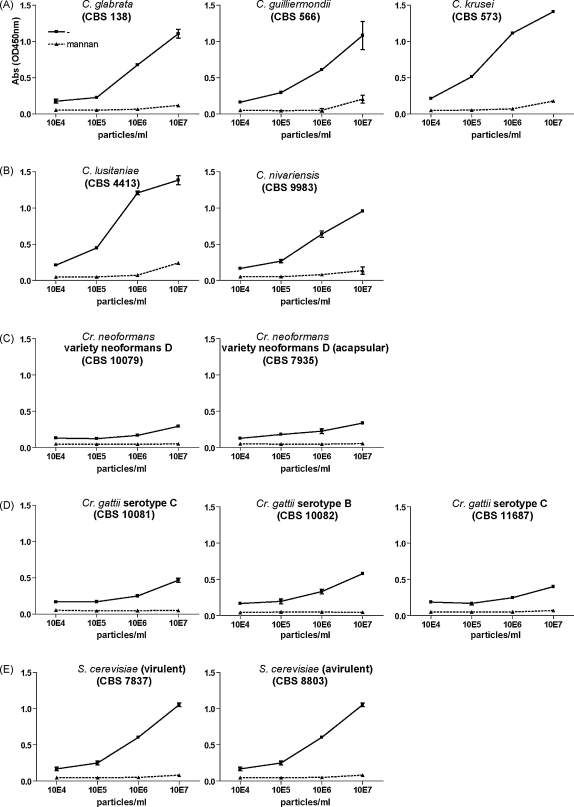
Titration of a selection of fungi. Yeast strains from *Candida* species (A and B), *Cryptococcus neoformans* (C), *Cryptococcus gattii* (D), and *Saccharomyces cerevisiae* (E) were coated onto ELISA plates at different concentrations. Mannan was used to determine specificity. Data are representative of at least three independent experiments. Error bars represent standard deviation of triplicates.

Langerin efficiently interacted with all *Candida* species tested, namely *C. albicans*, *C. glabrata*, *C. guilliermondii*, *C. krusei*, *C. lusitaniae*, and *C. nivariensis*, and *Saccharomyces cerevisiae* at a concentration of 10^6^ and 10^7^/ml, whereas most binding was lost at 10^5^/ml or lower (Figs. 1D and 2A, B and E). No difference was observed between virulent and non-virulent strains of *S. cerevisiae* (Fig. 2E). *Cryptococcus* species only weakly interacted with Langerin at high concentrations which was abrogated after dilution (Fig. 2C and D). These data demonstrate that Langerin is a fungal receptor that interacts with *Malassezia furfur*, *Saccharomyces* and *Candida* species but not with *Cryptococcus* species.

### 2.2 Langerin binds to β-glucans

**Fig. 3:**
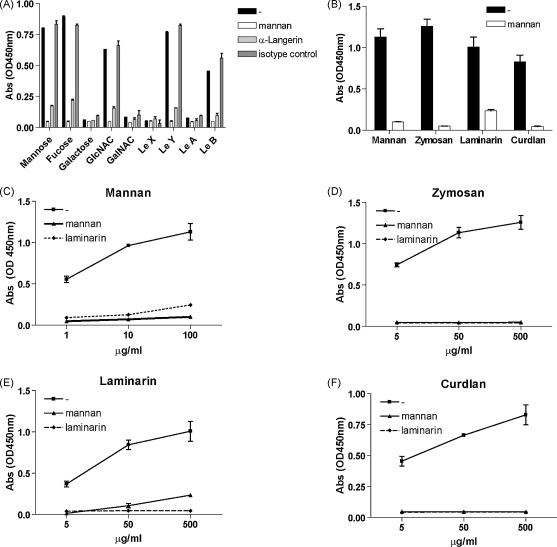
Langerin is a receptor for β-glucans. (A) Langerin specifically interacts with mannose, fucose and GlcNAc structures. Carbohydrate binding to Langerin was determined by recombinant Langerin ELISA. Specificity was determined by mannan, anti-Langerin (10E2) (de Witte et al., 2007) or isotype control antibodies. (B–F) Langerin is a receptor for β-glucans present on curdlan, laminarin and zymosan. Mannan (C), zymosan (D), laminarin (E) and curdlan (F) were coated onto ELISA plates in different concentrations. Binding to Langerin was inhibited by addition of mannan or laminarin. Data represent results from at least three independent experiments. Error bars represent standard deviation of triplicates.

Langerin has been shown to interact strongly with mannose, fucose and GlcNAc structures (Fig. 3A) (Stambach and Taylor, 2003). In addition, Langerin interacts with both Lewis B and Lewis Y through terminal fucose structures, whereas it does not interact with internal fucose present in Lewis A and Lewis X (de Jong et al., 2009). In general, fungal cell walls contain a large amount of mannose structures, chitin, as well as β(1–3) and β(1–6)-glucans at the inner-layer and budding scars (Chaffin et al., 1998). Mannan is a major component of the mannose structures on the cell wall and also a well-known ligand for Langerin (Stambach and Taylor, 2003; Chaffin et al., 1998). Therefore we investigated whether Langerin also interacts with other fungal-derived structures. Strikingly, Langerin strongly interacted with curdlan, laminarin and zymosan (Fig. 3B) and this binding was inhibited by both mannan and laminarin (3C–F). Both curdlan and laminarin contain large amount of β-glucans, while zymosan is a ghost cell derivative of *S. cerevisiae*, containing both mannose and β-glucans. It has previously been demonstrated that β-glucans partially inhibit zymosan binding to mouse Langerin, which suggests a role for Langerin in β-glucan binding (Takahara et al., 2004). Here, we demonstrate that Langerin directly interacts with β-glucans. These data demonstrate that Langerin recognizes also β-glucans, which could facilitate recognition of fungi.

### 2.3 Cellular Langerin interacts with *Candida* species and Zymosan but not with *Cryptococcus* species

**Fig. 4:**
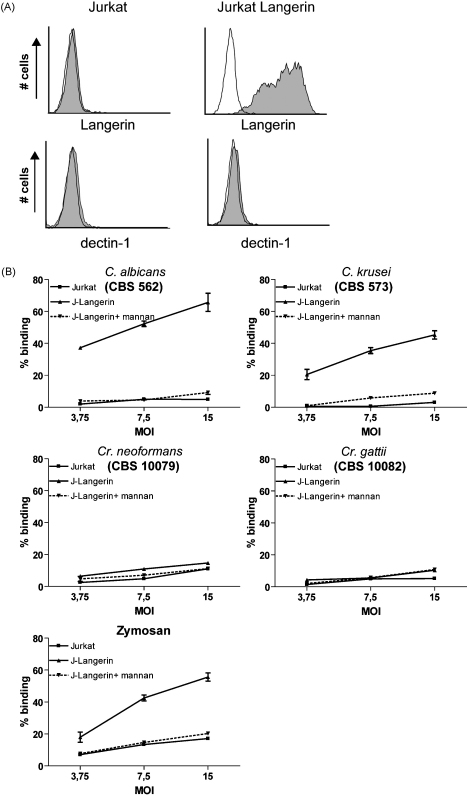
*Candida* species and zymosan interact with cellular Langerin. (A) Langerin and dectin-1 expression on Jurkat cell-line transduced with Langerin. Thin line represents isotype control, filled histogram represents specific staining. (B) Langerin expressing cell-line was incubated with different concentrations of FITC-labelled fungi and binding was measured by flow-cytometric analysis. Pre-incubation with mannan was used to determine specificity. Data represent results from at least three independent experiments. Error bars represent standard deviation of triplicates.

Next, we investigated whether a selection of this panel also interacted with cellular Langerin. Fluorescently labelled *C. albicans*, *C. krusei*, *Cr. neoformans*, *Cr. gattii*, and zymosan were incubated with a Langerin transduced cell-line, or a mock transduced cell-line. We selected the Jurkat T cell cell-line since it does not express dectin-1, which has been identified as a primary receptor for β-glucans (Fig. 4A). *C. albicans*, *C. krusei* and zymosan strongly interacted with Langerin transduced cells, which was completely inhibited by mannan, whereas no binding was observed to mock-transduced cells (Fig. 4B). In concordance with the Langerin binding ELISA, *Cr. neoformans* and *Cr. gattii* did neither interact with the Langerin transduced cells, nor with the mock-transduced cells. These data strongly suggest that Langerin recognizes *Candida* species but not *Cryptococcus* species.

### 2.4 Langerin is the primary receptor for fungi on primary Langerhans cells

**Fig. 5:**
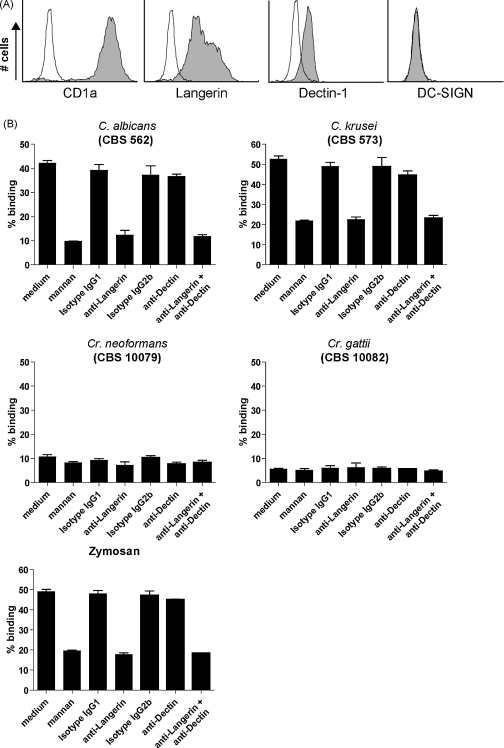
Langerin is the primary receptor for fungi on LCs. (A) CD1a, Langerin, dectin-1 and DC-SIGN expression on primary immature LCs. Thin line represents isotype control, filled histogram represents specific staining. (B) Binding of different fungi to human LCs was investigated. FITC-labelled fungi were incubated with LCs at MOI 10. Specificity was determined by mannan, blocking antibodies against Langerin and dectin-1, or isotype controls. Binding was measured by flow cytometry. Data represent results from at least three different donors. Error bars represent standard deviation of triplicates.

Opportunistic fungal infections often enter through the skin or mucosal tissues, where LCs are the predominant antigen presenting cells. Therefore, we investigated whether primary human LCs interact with different fungi. CD1a^+^ LCs isolated from human epidermis express high levels of Langerin, low levels of dectin-1 and no DC-SIGN (Fig. 5A). Both DC-SIGN and dectin-1 have been shown to bind fungi through mannan and β-glucan structures, respectively (Cambi et al., 2008; Brown et al., 2003). Primary LCs were incubated with fluorescently labelled *C. albicans*, *C. krusei*, *Cr. neoformans*, *Cr. gattii*, and zymosan and binding was measured by flow cytometry. Strikingly, LCs strongly interacted with *C. albicans*, *C. krusei*, and zymosan, but not with *Cr. neoformans* or *Cr. gattii* (Fig. 5B). The binding to *C. albicans*, *C. krusei*, and zymosan was primarily mediated via Langerin, since both mannan and specific antibodies against Langerin inhibited the binding to LCs (Fig. 5B). The blocking antibody against dectin-1 only weakly interfered with fungi binding to LCs, strongly suggesting that the contribution of dectin-1 on LCs is minor. *Cr. neoformans* and *Cr. gattii* failed to interact with LCs even at very high concentrations (data not shown). We observed a background binding of approximately 10%, which could be due to other receptors, such as complement receptor 3 expressed in low levels by LCs (Depanfilis et al., 1990; Netea et al., 2008).

**Fig. 6:**
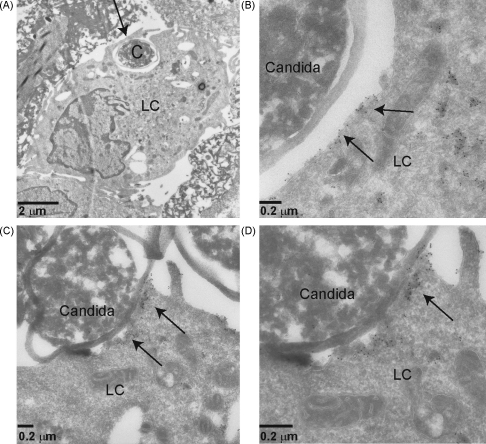
Electron microscopic analysis of *Candida albicans* interaction with LCs. (A) Electron microscopic picture of LCs which has internalized *C. albicans* (C). Arrow is directed towards *C. albicans*. (B–D) Gold labelling of Langerin demonstrates Langerin localization at the binding site of LCs and *C. albicans*. Arrows are directed towards Langerin gold labelling. Picture (B) is a magnification of (A), and (D) is a magnification of (C).

Next, we have used electron microscopy to investigate the uptake of *C. albicans* by LCs. LCs are able to phagocytose *C. albicans* (Fig. 6A), but we also observed *C. albicans* at the cell surface (Fig. 6). Notably, Langerin was regularly localized at the interface of the cell surface and *C. albicans*, either when phagocytosed or cell-bound (Fig. 6B–D). Irregular and detached cell wall was due to the EM preparation procedure. Thus, on LCs Langerin is the primary receptor for fungi such as *C. albicans* and the interaction can lead to phagocytosis of the fungi particles.

## 3 Discussion

Opportunistic fungal infections are a well-known problem, especially in immuno-compromised patients, such as HIV-1 infected people and transplantation patients. With an increase in the survival of these patients, there is also an increasingly large number of patients that suffer from opportunistic fungal infections (McNeil et al., 2001). *Candida* species, especially *C. albicans*, and *Cryptococcus* species are the most common causes of invasive fungal infections in immuno-compromised patients (Pappas et al., 2001, 2003). These fungi can colonize the skin and mucosal tissues that contain LCs and this dendritic cell subset might be crucial in the defense against fungi.

Our data show that Langerin is the major receptor on primary LCs for *Candida* species, *Saccharomyces* species, and *Malassezia furfur*. Notably, we did not observe any interaction of LCs with *Cryptococcus* species, suggesting that this pathogenic fungus is evading immune recognition by LCs.

Although *C. albicans* is still the leading cause of candidiasis, infections with variants such as *C. parapsilosis* and *C. glabrata* are increasingly common (Almirante et al., 2005; Arendrup et al., 2008). The cell wall of *Candida* species consists of mannose structures (mannan), chitins (GlcNAc structures) and β-1,3- and β-1,6-glucose polymers (β-glucans). The mannan components are present on the outer cell layer, whereas chitins and β-glucans are the structural components of the wall and exposed at budding scars (Karkowska-Kuleta et al., 2009). The C-type lectins dectin-1 and DC-SIGN are known fungal receptors that recognize fungi through β-glucan and mannan structures, respectively (Cambi et al., 2008). Here we demonstrate that Langerin is also a fungal receptor that interacts with a large variety of *Candida* species including *C. albicans*, *C. parapsilosis*, *C. glabrata*, *C. krusei*, *C. nivariensis*, *C. lusitaniae* and more. This suggests that *Candida* species express conserved structures that interact with Langerin. Strikingly, we observed that Langerin does not only recognizes mannose structures on fungi but also β1,3-glucan structures. Currently, dectin-1 is the main C-type lectin that specifically interacts with β-glucans and interaction of dectin-1 on macrophages and dendritic cells with *C. albicans* can modulate immune responses (Brown, 2006). Here, we demonstrate that Langerin also has affinity for β-glucans and that on human primary LCs, Langerin accounts for the majority of the interaction of *Candida* species. Together these data show that Langerin shares carbohydrate specificities with both DC-SIGN and dectin-1 (van Kooyk and Geijtenbeek, 2003; Brown, 2006).

The signalling pathways for dectin-1 and DC-SIGN have been largely identified and dectin-1 plays a central role in the defense against fungal infections (Brown, 2006; Geijtenbeek and Gringhuis, 2009). Since dectin-1 appears to play a minor role in the binding of *C. albicans* to LCs, and Langerin interaction is more prominent, it is of great interest to investigate the immunological consequences of Langerin binding to fungi. Using electron microscopy we observed co-localization of Langerin with *C. albicans*. We have previously demonstrated that interaction of viruses such as HIV-1 and Langerin can lead to internalization in Langerin positive Birbeck granules (de Witte et al., 2007). Due to the large size of *C. albicans* it seems unlikely that *C. albicans* can be internalized efficiently into Birbeck granules. However, our data demonstrate that LCs phagocytose fungi albeit not very efficiently. Questions remain whether Langerin is able to capture and degrade fungal pathogens in a similar manner as it is capable of degrading HIV-1. Noteworthy, we did observe that *C. albicans* but not *Cryptococcus* species could prevent the binding of HIV-1 to Langerin (data not shown), suggesting this could interfere with the protective barrier provides by Langerin on LCs and facilitate HIV-1 infection of LCs (de Witte et al., 2007). However, this remains to be further determined.

*S. cerevisiae* is one of the most well-studied yeast and shows high similarity to *C. albicans*’ cell-wall architecture (Karkowska-Kuleta et al., 2009). Indeed, similar to *C. albicans*, *S. cerevisiae* strongly interacted with Langerin. In addition, we observed comparable binding the non-virulent genome-sequenced strain of *S. cerevisiae* and a virulent strain. These data suggest that Langerin binding does not account for the differences in virulence.

*Malassezia* species are present as normal skin flora, however they are also associated with several skin diseases such as seborrehic dermatisis, atopic dermatitis, pityriasis versicolor and Malassezia folliculitis (Batra et al., 2005; Gupta et al., 2004). Most *Malassezia* species are lipid dependent (Xu et al., 2007), while one species, *M. pachydermatis*, is lipophilic and their cell wall is relatively thick (Mittag, 1995). Recently, it was demonstrated that *Malassezia* species have low mannose content in the cell wall, but strikingly *Malassezia* species reacted with anti-β-1,3-glucan antibody (Shibata et al., 2009). Here, we demonstrate that Langerin interacts with *M. furfur* and this suggests that the interaction is mediated via β-glucan structures. *M. furfur* and *M. pachydermatis* recently been identified as an activating ligand for C-type lectin Mincle (Yamasaki et al., 2009). Notably, we did not observe binding of Langerin to *M. pachydermatis* (data not shown). No difference in galactomannan spectra was observed by Shibata et al. (2009), however *M. pachydermatis* is a single non-lipid dependent species, which might account for the difference in binding to Langerin because of differences in cell-wall composition.

*Cryptococcus* species *Cr. neoformans*, which has a predilection for the central nervous system and is highly prevalent in HIV-1 infected patients, and *Cr. gattii*, which can cause infections in immune-competent patients, did not interact with Langerin. Although it has been described that the capsule of *Cr. neoformans* determines its virulence and allows immune escape by circumventing recognition by antigen presenting cells (Vecchiarelli et al., 2003; Kozubowski et al., 2009), binding by Langerin was not rescued using an acapsular variant. These data suggest that underneath the capsule, no carbohydrate structures are present that are recognized by Langerin. It remains to be determined whether acapsular *Cr. neoformans* can elicit proper immune response in LCs.

In this screening of opportunistic and pathogenic fungi interaction with Langerin and LCs we demonstrate that Langerin is a receptor for *Candida* species, *Saccharomyces* species and *Malassezia furfur* and we have shown that Langerin is a β-glucan receptor. This knowledge strongly suggests that Langerin is an important fungal receptor on LCs. It remains to be determined whether the binding is mediated via mannose structures or β-glucans present on fungi. Moreover, future studies are necessary to further examine the immunological consequences of these fungal interactions with Langerin on LCs and how this can contribute to the pathogenicity of fungal infections.

## 4 Materials and methods

### 4.1 Antibodies and reagents

The following antibodies and reagents were used: CD1a-FITC, Langerin-PE, DCGM4 (anti-Langerin) (all Immunotech), goat anti-mouse-FITC (Zymed Laboratories Inc.), isotype control IgG1 and IgG2b, anti dectin-1 (259931), and polyclonal anti-Langerin (all R&D systems), AZN-D1 (anti-DC-SIGN) (Geijtenbeek et al., 2000), 10E2 (anti-Langerin) (de Witte et al., 2007). Mannan from *S. cerevisiae*, curdlan from *Alcaligenes faecalis*, laminarin from *Laminaria digitata*, zymosan from *S. cerevisiae* (all Sigma–Aldrich), and zymosan-FITC (Molecular probes). Biotinylated polyacrylamide (PAA)-coupled glycoconjucates Mannose, Fucose, GlcNAc (N-acetyl-glucoseamine), GalNAc (N-acetyl-galactoseamine), Lewis (Le)X (Galβ1-4(Fucα1-3)GlcNAc), LeY (Fucα1-2Galβ1-4(Fucα1-3)GlcNAc), LeA (Galβ1-4(Fucα1-3)GlcNAc), LeB (Fucα1-2Galβ1-4(Fucα1-3)GlcNAc) were obtained from Lectinity. The following buffers were used: TSM buffer: Tris buffer (20 mM Tris–HCl, pH 7, 150 mM NaCl, 1 mM CaCl_2_, 2 mM MgCl_2_) (TSM), TSA buffer: TSM supplemented with BSA. PBA buffer: PBS supplemented with 0.5% BSA and 0.02% Azide.

### 4.2 Yeast preparation

Yeast strains were obtained from CBS Fungal Biodiversity Centre (www.cbs.knaw.nl) and included some new hybrid strains (Bovers et al., 2006, 2008). Yeast strains were cultured for 3 days at 25 °C on YPGA medium followed by inoculation in Sabouraud dextrose broth and incubated at 25 °C for 3 days, while shaking.

*Malassezia* strains were cultured on solid mLNA medium for 3 days at 30 °C. Yeast strains were harvested and heat inactivated for 1 h at 56 °C. Optical density measurement at 600 nm was used to determine the concentration. Yeast strains were washed extensively in PBS before storage at −20 °C at a concentration of 5 × 10^8^/ml until use.

### 4.3 Cell-lines and LC isolation

Jurkat and Jurkat-Langerin transduced cells were produced as described previously and were cultured in RPMI medium with 10% FCS (Valladeau et al., 2000; de Witte et al., 2007).

To isolate human LCs, human tissue was obtained from healthy donors undergoing corrective breast or abdominal surgery after informed consent in accordance with institutional guidelines and used within 3 h after surgery. The skin is cut in 0.3 mm slices containing the dermis and epidermis and incubated overnight at 4 °C in dispase II (1 mg/ml, Roche diagnostics) in Iscoves Modified Dulbecco's medium (IMDM) and gentamycin (10 μg/ml) overnight. The epidermis was mechanically separated and cultured in IMDM, 10% FCS, gentamycin (10 μg/ml), GM-CSF (800 U/ml) and IL-4 (800 U/ml). After 3 days the cells were harvested and layered on a ficoll density gradient to obtaining a 90% pure LC population as determined by flow cytometry and used immediately (de Witte et al., 2007).

### 4.4 Langerin binding ELISA

Different fungi (10^4^–10^7^/ml), carbohydrate structures (2 μg/ml) or β-glucans were coated onto ELISA plates overnight at room temperature. Non-specific binding was blocked by incubating the plate with 2% TSA buffer for 1 h at 37 °C. Recombinant human Langerin (Stambach and Taylor, 2003) (2 μg/ml in 2% TSA) was added for 1 h at 37 °C. Unbound Langerin was washed away and binding was determined using an anti-Langerin antibody (DCGM4, Beckman Coulter Inc.) followed by peroxidise-conjugated goat anti-mouse IgG antibody (Jackson Immunoresearch). Specificity was determined in the presence of mannan (1 mg/ml) or anti-Langerin monoclonal antibody 10E2 (10 μg/ml) (de Witte et al., 2007). Efficient coating of the different fungi was confirmed with plant-lectin binding ConA (*Canavalia ensiformis*).

### 4.5 Facs analysis

50,000 cells (Jurkat, Jurkat-Langerin or LCs) were washed in PBA and incubated with specific antibodies (5 μg/ml) or isotype controls for 30 min at 4 °C and followed by an incubation with FITC-labelled secondary antibodies for 30 min at 4 °C, or cells were incubated with directly labelled antibodies for 30 min at 4 °C. Cells were washed and binding was measured using flow cytometry.

### 4.6 Fitc labelling

Yeast strains were incubated with Fluorescein, 5′-isothiocyanate isomer I (FITC) (Sigma) (1 mg/ml in DMSO) for 1 h at room temperature. Yeast strains were washed extensively in PBS to eliminate unbound FITC. The solution was resuspended at a concentration of 1 × 10^8^/ml and stored at 4 °C in the dark until use.

### 4.7 Cell binding assay

50,000 cells (Jurkat, Jurkat-Langerin or LCs) were washed in 0.5% TSA and pre-incubated with mannan, blocking antibodies against Langerin or dectin-1, or isotype controls for 15 min at 37 °C. Different strains of FITC-labelled yeast were added for 45 min at 37 °C. Cells were washed and binding was measured using flow cytometry. Forward-site scatter gating was used to exclude unbound yeast.

### 4.8 Electron microscopy

LCs (1 × 10^6^) were incubated with 1 × 10^7^ *C. albicans* for 4 h. Cells were fixed and sections were prepared according to standard protocol (de Witte et al., 2007). Sections were stained for Langerin as described previously using polyclonal Langerin antibody (R&D) (de Witte et al., 2007).

## Figures and Tables

**Table 1:** Yeast strains and CBS numbers.

Strain	CBS no.	No.
*Candida albicans*	CBS 8781	1
*C. albicans*	CBS 562	2
*C. albicans*	CBS 8758	3
*C. albicans*	CBS 1912	4
*C. albicans*	CBS 2712	5
*C. albicans*	CBS 5144	6
*C. dubliniensis*	CBS 8501	7
*C. dubliniensis*	CBS 8500	8
*C. glabrata*	CBS 138	9
*C. glabrata*	CBS 860	10
*C. guilliermondii*	CBS 566	11
*C. guilliermondii*	CBS 2031	12
*C. krusei*	CBS 573	13
*C. parapsilosis*	CBS 604	14
*C. parapsilosis*	CBS 2195	15
*C. tropicalis*	CBS 94	16
*C. tropicalis*	CBS 1920	17
*C. lusitaniae*	CBS 4413	18
*C. lusitaniae*	CBS 4414	19
*C. nivariensis*	CBS 9983	20
*C. nivariensis*	CBS 9985	21
*C. orthopsilosis*	CBS 10741	22
*C. orthopsilosis*	CBS 10906	23
*C. metapsilosis*	CBS 10746	24
*C. metapsilosis*	CBS 10747	25
*Malassezia furfur*	CBS 1878	26
*Cryptococcus neoformans variety grubii* serotype A	CBS 10085	27
*Cr. neoformans variety grubii* serotype A	CBS 10083	28
*Cr. neoformans variety neoformans* serotype D	CBS 10079	29
*Cr. neoformans variety neoformans* serotype D (acapsular)	CBS 7935	30
*Cr. neoformans variety neoformans* serotype AD hybrid	CBS 10080	31
*Cr. gattii* serotype B	CBS 10078	32
*Cr. gattii* serotype C	CBS 10081	33
*Cr. gattii* serotype B	CBS 10082	34
*Cr. gattii* serotype C	CBS 10101	35
*Cr. gattii* serotype B	CBS 11687	36
*Cr. neoformans* × *Cr. gattii* serotype BD hybrid	CBS 10488	37
*Cr. neoformans* × *Cr. gattii* serotype AB hybrid	CBS 10496	38
*Saccharomyces cerevisiae* (virulent)	CBS 7837	39
*Saccharomyces cerevisiae* (avirulent, genome sequenced strain)	CBS 8803	40

## References

[bib1] Almirante B., Rodriguez D., Park B.J., Cuenca-Estrella M., Planes A.M., Almela M., Mensa J., Sanchez F., Ayats J., Gimenez M., Saballs P., Fridkin S.K., Morgan J., Rodriguez-Tudela J.L., Warnock D.W., Pahissa A. (2005). Epidemiology and predictors of mortality in cases of *Candida* bloodstream infection: results from population-based surveillance, barcelona, Spain, from 2002 to 2003. J. Clin. Microbiol..

[bib2] Arendrup M.C., Fuursted K., Gahrn-Hansen B., Schonheyder H.C., Knudsen J.D., Jensen I.M., Bruun B., Christensen J.J., Johansen H.K. (2008). Semi-national surveillance of fungaemia in Denmark 2004–2006: increasing incidence of fungaemia and numbers of isolates with reduced azole susceptibility. Clin. Microbiol. Infect..

[bib3] Banchereau J., Steinman R.M. (1998). Dendritic cells and the control of immunity. Nature.

[bib4] Batra R., Boekhout T., Gueho E., Cabanes F.J., Dawson T.L., Gupta A.K. (2005). *Malassezia Baillon*, emerging clinical yeasts. FEMS Yeast Res..

[bib5] Bovers M., Hagen F., Kuramae E.E., Diaz M.R., Spanjaard L., Dromer F., Hoogveld H.L., Boekhout T. (2006). Unique hybrids between the fungal pathogens *Cryptococcus neoformans* and *Cryptococcus gattii*. FEMS Yeast Res..

[bib6] Bovers M., Hagen F., Kuramae E.E., Hoogveld H.L., Dromer F., St-Germain G., Boekhout T. (2008). AIDS patient death caused by novel *Cryptococcus neoformans* × *C. gattii* hybrid. Emerg. Infect. Dis..

[bib7] Brown G.D. (2006). Dectin-1: a signalling non-TLR pattern-recognition receptor. Nat. Rev. Immunol..

[bib8] Brown G.D., Gordon S. (2001). Immune recognition. A new receptor for beta-glucans. Nature.

[bib9] Brown G.D., Herre J., Williams D.L., Willment J.A., Marshall A.S., Gordon S. (2003). Dectin-1 mediates the biological effects of beta-glucans. J. Exp. Med..

[bib10] Cambi A., Netea M.G., Mora-Montes H.M., Gow N.A., Hato S.V., Lowman D.W., Kullberg B.J., Torensma R., Williams D.L., Figdor C.G. (2008). Dendritic cell interaction with *Candida albicans* critically depends on N-linked mannan. J. Biol. Chem..

[bib11] Chaffin W.L., Lopez-Ribot J.L., Casanova M., Gozalbo D., Martinez J.P. (1998). Cell wall and secreted proteins of *Candida albicans*: identification, function, and expression. Microbiol. Mol. Biol. Rev..

[bib12] de Jong M.A., de Witte L., Santegoets S.J., Fluitsma D., Taylor M.E., de Gruijl T.D., Geijtenbeek T.B. (2009). Mutz-3-derived Langerhans cells are a model to study HIV-1 transmission and potential inhibitors. J. Leukoc. Biol..

[bib13] de Witte L., Nabatov A., Pion M., Fluitsma D., de Jong M.A., de Gruijl T.D., Piguet V., van Kooyk Y., Geijtenbeek T.B. (2007). Langerin is a natural barrier to HIV-1 transmission by Langerhans cells. Nat. Med..

[bib14] Depanfilis G., Soligo D., Manara G.C., Ferrari C., Torresani C., Zucchi A. (1990). Human normal-resting epidermal langerhans cells do express the type-3 complement receptor. Br. J. Dermatol..

[bib15] Fithian E., Kung P., Goldstein G., Rubenfeld M., Fenoglio C., Edelson R. (1981). Reactivity of Langerhans cells with hybridoma antibody. Proc. Natl. Acad. Sci. U.S.A..

[bib16] Gantner B.N., Simmons R.M., Underhill D.M. (2005). Dectin-1 mediates macrophage recognition of *Candida albicans* yeast but not filaments. EMBO J..

[bib17] Geijtenbeek T.B., Gringhuis S.I. (2009). Signalling through C-type lectin receptors: shaping immune responses. Nat. Rev. Immunol..

[bib18] Geijtenbeek T.B., Torensma R., van Vliet S.J., van Duijnhoven G.C., Adema G.J., van Kooyk Y., Figdor C.G. (2000). Identification of DC-SIGN, a novel dendritic cell-specific ICAM-3 receptor that supports primary immune responses. Cell.

[bib19] Gupta A.K., Batra R., Bluhm R., Boekhout T., Dawson T.L. (2004). Skin diseases associated with *Malassezia* species. J. Am. Acad. Dermatol..

[bib20] Hunger R.E., Sieling P.A., Ochoa M.T., Sugaya M., Burdick A.E., Rea T.H., Brennan P.J., Belisle J.T., Blauvelt A., Porcelli S.A., Modlin R.L. (2004). Langerhans cells utilize CD1a and Langerin to efficiently present nonpeptide antigens to T cells. J. Clin. Invest..

[bib21] Karkowska-Kuleta J., Rapala-Kozik M., Kozik A. (2009). Fungi pathogenic to humans: molecular bases of virulence of *Candida albicans*, *Cryptococcus neoformans* and *Aspergillus fumigatus*. Acta Biochim. Pol..

[bib22] Kozubowski L., Lee S.C., Heitman J. (2009). Signalling pathways in the pathogenesis of Cryptococcus. Cell Microbiol..

[bib23] McNeil M.M., Nash S.L., Hajjeh R.A., Phelan M.A., Conn L.A., Plikaytis B.D., Warnock D.W. (2001). Trends in mortality due to invasive mycotic diseases in the United States, 1980–1997. Clin. Infect. Dis..

[bib24] Mittag H. (1995). Fine structural investigation of *Malassezia furfur*. II. The envelope of the yeast cells. Mycoses.

[bib25] Netea M.G., Brown G.D., Kullberg B.J., Gow N.A.R. (2008). An integrated model of the recognition of *Candida albicans* by the innate immune system. Nat. Rev. Microbiol..

[bib26] Pappas P.G., Perfect J.R., Cloud G.A., Larsen R.A., Pankey G.A., Lancaster D.J., Henderson H., Kauffman C.A., Haas D.W., Saccente M., Hamill R.J., Holloway M.S., Warren R.M., Dismukes W.E. (2001). Cryptococcosis in human immunodeficiency virus-negative patients in the era of effective azole therapy. Clin. Infect. Dis..

[bib27] Pappas P.G., Rex J.H., Lee J., Hamill R.J., Larsen R.A., Powderly W., Kauffman C.A., Hyslop N., Mangino J.E., Chapman S., Horowitz H.W., Edwards J.E., Dismukes W.E. (2003). A prospective observational study of candidemia: epidemiology, therapy, and influences on mortality in hospitalized adult and pediatric patients. Clin. Infect. Dis..

[bib28] Patterson B.K., Landay A., Siegel J.N., Flener Z., Pessis D., Chaviano A., Bailey R.C. (2002). Susceptibility to human immunodeficiency virus-1 infection of human foreskin and cervical tissue grown in explant culture. Am. J. Pathol..

[bib29] Richardson M.D. (2005). Changing patterns and trends in systemic fungal infections. J. Antimicrob. Chemother..

[bib30] Shibata N., Saitoh T., Tadokoro Y., Okawa Y. (2009). The cell wall galactomannan antigen from *Malassezia furfur* and *Malassezia pachydermatis* contains beta-1,6-linked linear galactofuranosyl residues and its detection has diagnostic potential. Microbiology.

[bib31] Stambach N.S., Taylor M.E. (2003). Characterization of carbohydrate recognition by Langerin, a C-type lectin of Langerhans cells. Glycobiology.

[bib32] Takahara K., Omatsu Y., Yashima Y., Maeda Y., Tanaka S., Iyoda T., Clusen B., Matsubara K., Letterio J., Steinman R.M., Matsuda Y., Inaba K. (2002). Identification and expression of mouse Langerin (CD207) in dendritic cells. Int. Immunol..

[bib33] Takahara K., Yashima Y., Omatsu Y., Yoshida H., Kimura Y., Kang Y.S., Steinman R.M., Park C.G., Inaba K. (2004). Functional comparison of the mouse DC-SIGN, SIGNR1, SIGNR3 and Langerin, C-type lectins. Int. Immunol..

[bib34] Turville S.G., Arthos J., Mac Donald K., Lynch G., Naif H., Clark G., Hart D., Cunningham A.L. (2001). HIV gp120 receptors on human dendritic cells. Blood.

[bib35] Valladeau J., Ravel O., Dezutter-Dambuyant C., Moore K., Kleijmeer M., Liu Y., Duvert-Frances V., Vincent C., Schmitt D., Davoust J., Caux C., Lebecque S., Saeland S. (2000). Langerin, a novel C-type lectin specific to Langerhans cells, is an endocytic receptor that induces the formation of Birbeck granules. Immunity.

[bib36] Valladeau J., Zutter-Dambuyant C., Saeland S. (2003). Langerin/CD207 sheds light on formation of birbeck granules and their possible function in Langerhans cells. Immunol. Res..

[bib37] van Kooyk Y., Geijtenbeek T.B. (2003). DC-SIGN: escape mechanism for pathogens. Nat. Rev. Immunol..

[bib38] Vecchiarelli A., Pietrella D., Lupo P., Bistoni F., McFadden D.C., Casadevall A. (2003). The polysaccharide capsule of *Cryptococcus neoformans* interferes with human dendritic cell maturation and activation. J. Leukoc. Biol..

[bib39] Xu J., Saunders C.W., Hu P., Grant R.A., Boekhout T., Kuramae E.E., Kronstad J.W., Deangelis Y.M., Reeder N.L., Johnstone K.R., Leland M., Fieno A.M., Begley W.M., Sun Y., Lacey M.P., Chaudhary T., Keough T., Chu L., Sears R., Yuan B., Dawson T.L. (2007). Dandruff-associated Malassezia genomes reveal convergent and divergent virulence traits shared with plant and human fungal pathogens. Proc. Natl. Acad. Sci. U.S.A..

[bib40] Yamasaki S., Matsumoto M., Takeuchi O., Matsuzawa T., Ishikawa E., Sakuma M., Tateno H., Uno J., Hirabayashi J., Mikami Y., Takeda K., Akira S., Saito T. (2009). C-type lectin Mincle is an activating receptor for pathogenic fungus, Malassezia. Proc. Natl. Acad. Sci. U.S.A..

